# Development of a whole-cell SELEX process to select species-specific aptamers against *Aspergillus niger*

**DOI:** 10.1186/s40694-024-00185-2

**Published:** 2024-11-05

**Authors:** Valeria Ellena, Alexandra Ioannou, Claudia Kolm, Andreas H. Farnleiter, Matthias G. Steiger

**Affiliations:** 1 Research Group Biochemistry, Institute of Chemical, Environmental and Bioscience Engineering, TU Wien, Vienna, Austria; 2 Austrian Centre of Industrial Biotechnology (ACIB GmbH), Muthgasse 18, Vienna, Austria; 3 Department Pharmacology, Physiology and Microbiology, Division of Waterquality and Health, Karl Landsteiner University of Health Sciences, Dr. -Karl-Dorrek-Straße 30, Krems, Austria; 4 Research Group Microbiology and Molecular Diagnostics, Institute of Chemical, Environmental and Bioscience Engineering, TU Wien, Vienna, Austria

**Keywords:** Biosensors, Fungal conidia, DNA aptamers, Spores, Aspergillosis

## Abstract

**Background:**

Spores produced by the filamentous fungus *Aspergillus niger* are abundant in a variety of environments. The proliferation of this fungus in indoor environments has been associated to health risks and its conidia can cause allergic reaction and severe invasive disease in animals and humans. Therefore, the detection and monitoring of *Aspergillus* conidia is of utmost importance to prevent serious fungal infections and contaminations. Among others, aptamers could serve as biosensors for the specific detection of fungal spores.

**Results:**

In this study, DNA aptamers specific to conidia of *A. niger* were developed by optimizing a whole-cell SELEX approach. Three whole-cells SELEX experiments were performed in parallel with similar conditions. Quantification of recovered ssDNA and melting curve analyses were applied to monitor the ongoing SELEX process. Next-generation sequencing was performed on selected recovered ssDNA pools, allowing the identification of DNA aptamers which bind with high affinity to the target cells. The developed aptamers were shown to be species-specific, being able to bind to *A. niger* but not to *A. tubingensis* or to *A. nidulans*. The binding affinity of two aptamers (AN01-R9-006 and AN02-R9-185) was measured to be 58.97 nM and 138.71 nM, respectively, which is in the range of previously developed aptamers.

**Conclusions:**

This study demonstrates that species-specific aptamers can be successfully developed via whole-cell SELEX to distinguish different *Aspergillus* species and opens up new opportunities in the field of diagnostics of fungal infections.

**Supplementary Information:**

The online version contains supplementary material available at 10.1186/s40694-024-00185-2.

## Introduction

*Aspergillus niger* is a biotechnologically relevant filamentous fungus widely utilized in industry for its outstanding capability of producing organic acids and enzymes [5, 6]. Bulk chemicals and proteins produced by this fungus are regarded as safe by the regulatory authorities and find applications in a variety of commodity products. Generally, *A. niger* is regarded as non-toxic and non-pathogenic for healthy individuals but it has the potential to cause allergic reactions and infectious diseases in patients with a compromised immune system [11, 20, 23]. Aspergillosis, in particular, poses a serious threat as it can lead to a fatal outcome if not diagnosed promptly.

Besides, some strains of *A. niger* are able to produce mycotoxins, such as fumonisins, ochratoxin A and oxalic acid [11], which can contaminate food products and cause multiple diseases in animals and humans [11, 28]. *A. niger* is ubiquitous, being able to grow at a wide range of temperatures and pHs, and its conidia can be found in various natural as well as in indoor environments [23]. Monitoring and diagnostics of *A. niger* conidia is crucial to prevent and manage spread of fungal infections and contamination of food products and air. The detection and identification of *Aspergillus* conidia currently depend on sample collection followed by culturing, microscopy and metabolite, serological or molecular analyses. These methods are often time-consuming, lack sensitivity or require sophisticated equipment [21]. Additionally, unless a molecular analysis is performed, they do not provide information on the specific species involved. Even antibodies against *Aspergillus* tend to have broad specificity and are unable to distinguish between species. However, rapid identification at the species level is crucial in the case of aspergillosis, as it allows to provide the most effective treatment to the patient.

Therefore, a rapid and species-specific biosensor for *A. niger* conidia would facilitate the detection of fungal spores in critical environments and even support the early diagnosis of a fungal infection in a hospital setting [13].

Species-specific detection of fungal conidia could be achieved using specific aptamers. Aptamers are short (typically less than 100 k-mer) single-stranded oligonucleotide (DNA or RNA) molecules that, upon folding into a tridimensional structure, can bind with high affinity to any target of interest against which they were selected. They can potentially be developed starting from a random oligonucleotide library against various target of interest, from small molecules to proteins, with an iterative process called SELEX (Systematic evolution of ligands by exponential enrichment)[9, 31]. Even more complex targets can be used [15, 19], such as entire cells, in which case the process is termed whole-cell SELEX [25]. Once identified, aptamers can be readily modified with fluorophores or chemical groups for multiple applications [37]. From an industrial standpoint, DNA aptamers can be synthesized at large-scale, outperforming antibodies in terms of costs, batch-to-batch reproducibility and stability of the final product [37]. Next to the potential diagnostic applications of *Aspergillus*-specific aptamers, these molecules could help understand the composition of the fungal conidial surface. The outer layer of conidia, the cell wall, mainly consists of proteins and polysaccharides and plays an important role in the interaction of the conidia with the external environment [12]. The particular composition of the conidial wall is not only crucial for the establishment of an infection but can also have significance for industrial applications [4]. For example, pellet morphology, an important prerequisite for production of citric acid with *A. niger*, is strongly affected by the cell wall composition [38]. Therefore, investigating the presence or the absence of specific surface constituents can be beneficial for multiple applications.

Successful implementation of whole-cell SELEX was already shown against various microorganisms, including bacteria [30] and yeast [2]. More recently, whole-cell SELEX was applied against spores produced by fungi. Krivitsky and colleagues developed an electrochemical aptamer-based method to collect and detect spores produced by the basidiomycete plant pathogen *Phakopsora pachyrhizi* [18]. Aptamers recognizing three different species of *Aspergillus* (*A. fumigatus*, *A. flavus* and *A. niger*) were developed. These aptamers were obtained by subsequent incubation of the recovered ssDNA with the three fungal species, so that the enriched sequences were not selected for species-specificity [26].

In this study, we focused on the identification and selection of aptamers able to recognize *A. niger* conidia at the species level. To this end, a whole-cell SELEX approach was optimized by combining two previously published protocols [17, 26]. Differently from the approach of Seo and colleagues, a counter-selection step with *A. tubingensis* was introduced in the SELEX protocol, leading to the identification and selection of species-specific aptamers against *A. niger* conidia over three independent whole-cell SELEX experiments.

## Materials and methods

### Buffers and chemicals

10 × PBST (1.37 M NaCl, 27 mM KCl, 100 mM Na_2_HPO_4_ × 2H_2_O, 18 mM KH_2_PO_4_ and 0.5% Tween20, pH 7.4) was prepared as stock solution, filtered and stored at room temperature.

1 × PBST was prepared by diluting 10 × PBST with sterile ultrapure lab water prepared with Milli-Q system (Merck) and stored at 4 °C.

10 × MgCl_2_ (14 mM) was prepared in 1 × PBST and stored at −20 °C.

Binding buffer was prepared fresh by diluting MgCl_2_ in 1 × PBST (1.4 mM final MgCl_2_ concentration).

BSA or recombinant albumin (NEB) and salmon sperm DNA (Thermo Fisher Scientific) were used as competitors at a final concentration of 0.5 and 0.25 µg/µL, respectively.

### Strains and culture conditions

Conidia of the *A. niger* strains ATCC 1015 and CBS 544.65 were used concomitantly as target for the whole-cell SELEX process, to increase the likelihood of selecting aptamers able of recognizing targets common across different strains. These two strains were selected because, while still belonging to the same species, they are classified in different clades [24]. Conidia of *A. tubingensis* MA 3973 (ACBR Fungal Database: (https://acbr-database.boku.ac.at) [8]) were used for counter-selection, due to the close phylogenetic proximity of *A. tubingensis* to *A. niger*.

Conidia of *A. tubingensis* MA 3973 and of *A. nidulans* FGSC A4 were used for species-specificity characterizations.

Strains were inoculated from glycerol stocks on minimal medium plates [3] and incubated for 5 days at 30 °C. Conidia were harvested from the plates with 0.1% Tween20, washed twice with 1 × PBST (5,000 rpm and 10 min) and resuspended in 1 × PBST. Conidia concentration was determined using a Thoma counting chamber.

### ssDNA library and primers

The random ssDNA library consisted of a randomized region of 40 nucleotides flanked by 23 constant primer binding sites (5´-TAGGGAAGAGAAGGACATATGAT-N_40_-TTGACTAGTACATGACCACTTGA-3`). It was ordered from IDT (Coralville, USA) with unique handmix ratio of the random bases and HPLC purification.

Modified primers 5´-/56-FAM/TAGGGAAGAGAAGGACATATGAT-3´and 5´-/5Phosph/TCAAGTGGTCATGTACTAGTCAA-3´ were used for the amplification of the recovered ssDNA pool after each SELEX round.

Unmodified primers (5´-TAGGGAAGAGAAGGACATATGAT-3´ and 5´-TCAAGTGGTCATGTACTAGTCAA-3´) were used for DNA quantification, melting curve analyses and characterization studies.

A random but specific 40 bp sequence flanked by the common primer binding sites (BA-NC-1: 5´-TAGGGAAGAGAAGGACATATGATGCTAGATGGACTTGCCGTTGGAAGACACAGCATGACCCCGTTGACTAGTACATGACCACTTGA-3´) was used as negative control for the binding assays. Two additional random and specific 40 bp sequences flanked by the common primer binding sites (BA-NC-2: 5´-TAGGGAAGAGAAGGACATATGATTACCTATCGCCTGAAAGCCAGTTGGTGTTAAGGAGTGCTCTTGACTAGTACATGACCACTTGA-3´and BA-NC-3: 5´- TAGGGAAGAGAAGGACATATGATAGCGCTCCCAGCACAACGGCCAAGGAAGTCTCCAATTTCTTTGACTAGTACATGACCACTTGA-3´) were used for specificity tests based on fluorescent measurements.

Primers, selected candidate aptamers and the negative control sequence were ordered from IDT (Coralville, USA) purified by standard desalting. Library, primers and aptamers were resuspended and diluted in ultrapure nuclease-free water to a final concentration of 100 µM.

### Whole-cell SELEX

Three independent whole-cell SELEX experiments (SELEX−1, −2 and −3) consisting of 9 consecutive rounds were performed according to a previously published protocol [17] with some modifications. Conidia suspensions were prepared in PBST as described above. 5 × 10^6^ conidia of each *A. niger* strain (ATCC 1015 and CBS 554.65) were pipetted in the same tube, centrifuged at 5000 g for 5 min and resuspended in the appropriate volume of binding buffer (Tables 1, 2 and 3). The ssDNA library (in the first round) or the recovered ssDNA pool was incubated for 1 h at room temperature in binding buffer in a total volume of 50 µL and then added to the resuspended conidia. Reaction volume and ssDNA concentration were specific for each round (Tables 1, 2 and 3). Incubation was performed in a thermoblock at 21 °C for 30 min and 650 rpm. Additionally, samples were shaken by hand every 5 min to avoid settling of the conidia to the bottom of the tube. After incubation, samples were washed with 1 mL binding buffer and resuspended in 50 µL ultrapure nuclease-free water. The number of washes was gradually increased over the rounds (Tables 1, 2 and 3). To allow elution of the aptamers from the conidia, the samples were incubated for 10 min at 95 °C, followed by 10 min on ice. They were then centrifuged at 5,000 g for 5 min and the supernatant was transferred to fresh tubes to which 0.1 volumes of 3 M sodium acetate and 3 volumes of 96% ethanol were added for overnight precipitation at −20 °C.

**Table 1:** Selection conditions applied in SELEX-1

Whole-cell SELEX-1								
Round	ssDNA		Positive selection		Reaction volume (µL)	Competitors	Washes	Counter-selection
	pmol	Final conc (nM)	Target strains	Number of conidia				
1	1800	7200	ATCC 1015, CBS 554.65	10^7^	250	–	1	–
2	25	100	ATCC 1015, CBS 554.65	10^7^	250	–	1	–
3	10	100	ATCC 1015, CBS 554.65	10^7^	100	BSA, 4sDNA	2	–
4	10	100	ATCC 1015, CBS 554.65	10^7^	100	–	2	*A. tubingensis* MA 3973
5	10	100	ATCC 1015, CBS 554.65	10^7^	100	BSA, 4sDNA	3	*A. tubingensis* MA 3973
6	10	100	ATCC 1015, CBS 554.65	10^7^	100	BSA, 4sDNA	3	*A. tubingensis* MA 3973
7	10	100	ATCC 1015, CBS 554.65	10^7^	100	BSA, 4sDNA	3	*A. tubingensis* MA 3973
8	10	100	ATCC 1015, CBS 554.65	10^7^	100	BSA, 4sDNA	5	*A. tubingensis* MA 3973
9-N	10	100	ATCC 1015, CBS 554.65	10^7^	100	BSA, 4sDNA	6	–
9-T	10	100	*A. tubingensis* MA 3973	10^7^	100	BSA, 4sDNA	6	–

**Table 2:** Selection conditions applied in SELEX-2

Whole-cell SELEX-2								
Round	ssDNA		Positive selection		Reaction volume (µL)	Competitors	Washes	Counter-selection
	pmol	Final conc (nM)	Target strains	Number of conidia				
1	1800	7200	ATCC 1015, CBS 554.65	10^7^	250	–	1	–
2	25	100	ATCC 1015, CBS 554.65	10^7^	250	–	1	–
3	25	100	ATCC 1015, CBS 554.65	10^7^	250	BSA, 4sDNA	2	–
4	10	100	ATCC 1015, CBS 554.65	10^7^	100	–	2	*A. tubingensis* MA 3973
5	8	100	ATCC 1015, CBS 554.65	10^7^	80	BSA, 4sDNA	3	*A. tubingensis* MA 3973
6	8	100	ATCC 1015, CBS 554.65	10^7^	80	BSA, 4sDNA	3	*A. tubingensis* MA 3973
7	8	100	ATCC 1015, CBS 554.65	10^7^	80	BSA, 4sDNA	3	*A. tubingensis* MA 3973
8	8	80	ATCC 1015, CBS 554.65	10^7^	100	BSA, 4sDNA	6	*A. tubingensis* MA 3973
9-N	8	80	ATCC 1015, CBS 554.65	10^7^	100	BSA, 4sDNA	6	–
9-T	8	80	*A. tubingensis* MA 3973	10^7^	100	BSA, 4sDNA	6	–

**Table 3:** Selection conditions applied in SELEX-3

Whole-cell SELEX-3								
Round	ssDNA		Positive selection		Reaction volume (µL)	Competitors	Washes	Counter-selection
	pmol	Final conc (nM)	Target strains	Number of conidia				
1	1800	7200	ATCC 1015, CBS 554.65	10^7^	250	–	1x	–
2	25	100	ATCC 1015, CBS 554.65	10^7^	250	BSA, 4sDNA	1x	–
3	23	92	ATCC 1015, CBS 554.65	10^7^	250	BSA, 4sDNA	2x	–
4	23	92	ATCC 1015, CBS 554.65	10^7^	250	BSA, 4sDNA	2x	*A. tubingensis* MA 3973
5	9.2	92	ATCC 1015, CBS 554.65	10^7^	100	BSA, 4sDNA	3x	*A. tubingensis* MA 3973
6*	9.2	92	ATCC 1015, CBS 554.65	10^7^	100	BSA, 4sDNA	3x	*A. tubingensis* MA 3973
7	9.2	92	ATCC 1015, CBS 554.65	10^7^	100	BSA, 4sDNA	3x	*A. tubingensis* MA 3973
8	9.2	92	ATCC 1015, CBS 554.65	10^7^	100	BSA, 4sDNA	5x	*A. tubingensis* MA 3973
9	9.2	92	ATCC 1015, CBS 554.65	10^7^	100	BSA, 4sDNA	5x	*A. tubingensis* MA 3973
9-N	9.2	92	ATCC 1015, CBS 554.65	10^7^	100	BSA, 4sDNA	5x	–
9-T	9.2	92	*A. tubingensis* MA 3973	10^7^	100	BSA, 4sDNA	5x	–

*ssDNA applied to round 6 was derived from a mixture of dsDNA obtained from the amplification of the ssDNA recovered after round 5 and amplified from the already amplified ssDNA

Precipitated ssDNA was recovered by centrifugation (20 min, 16,000 g, 4 °C) and washed twice with 70% ethanol. It was then resuspended in 50 µL ultrapure nuclease-free water.

Recovered ssDNA pools were amplified by PCR. To determine the optimal number of cycles for the enrichment PCR, two test-PCRs were performed (dnc1 and dnc2).

All the ssDNA recovered after the first cycle (50 µL) was first amplified with 6 cycles and subsequently purified to allow enrichment of each of the recovered sequences before proceeding with the test-PCRs. To enrich the ssDNA recovered after the first round, 10 reactions of 25 µL were set up, each containing 5 µL of the recovered ssDNA, 1 × Q5 buffer (NEB), 0.2 mM dNTPs, 1 µM of each modified primer and 0.5 units of Q5 High-Fidelity DNA polymerase (NEB).

Dnc1 was performed in a reaction volume of 25 µL containing 1 µL of recovered ssDNA (purified PCR product for round 1), 1 × Q5 buffer (NEB), 0.2 mM dNTPs, 1 µM of each modified primer, 1 × EvaGreen Plus dye (Biotium) and 0.5 units of Q5 High-Fidelity DNA polymerase (NEB). The thermocycling program was the following: denaturation for 3 min at 95 °C followed by 30 cycles of 15 s at 95 °C, 15 s at 58 °C and 15 s at 72 °C, and final elongation for 2 min at 72 °C. Fluorescence was acquired at each cycle on the green channel during the first elongation step. The amplification range was determined based on the amplification curve obtained from the fluorescence measurement and three cycles before the peak were selected for dnc2.

Dnc2 was performed with the same conditions of dnc1 but without EvaGreen dye. Three reactions were set up in parallel and stopped at different number of cycles. Aliquots of the PCR products were loaded on a 3% agarose gel stained with SYBR Gold (Thermo Fisher Scientific) and the optimal number of cycles was selected based on robust amplification without by-product/heteroduplex formation. ssDNA (22–25 reactions) was then amplified with the same conditions used for dnc2. Amplified DNA was purified with the Monarch PCR & DNA Cleanup Kit from NEB and digested to ssDNA with a Lambda Exonuclease (NEB).

For ssDNA generation, multiple 50 µL reactions containing 500 ng of DNA, 1 × Lambda exonuclease reaction buffer and 0.5 units of Lambda Exonuclease were prepared. Samples were incubated at 37 °C for 30 min, followed by enzyme inactivation at 80 °C for 10 min. Aliquots of the generated ssDNA were checked on a 4% agarose gel stained with SYBR Gold (Thermo Fisher Scientific) and the samples were purified with the Oligonucleotide Cleanup protocol included in the Monarch PCR & DNA Cleanup Kit from NEB. An aliquot of the generated ssDNA was checked on a 4% agarose gel and the concentration determined at the NanoDrop. Over the rounds, more stringent conditions (decreasing the starting DNA concentration, increasing the number of washes and addition of competitors during the incubation) were applied as reported in Tables 1, 2 and 3.

Counter-selection was performed in rounds 4 to 8 with *A. tubingensis*. In brief, recovered, amplified and single-stranded generated DNA was incubated with 10^7^ *A. tubingensis* conidia for 30 min at 21 °C. The samples were then centrifuged and the supernatant containing unbound sequences was added to *A. niger* conidia before continuing with the standard protocol.

In round 9, a negative selection (R9-T) was performed in parallel with the standard protocol (R9-N). In this case, the same protocol was applied, with the difference that conidia of *A. tubingensis* were used as target with the aim of identifying and excluding aptamer sequences not species-specific for *A. niger*.

### Quantification of recovered ssDNA by qPCR

qPCR was performed using a RotorGene Q (Qiagen) in a total volume of 15 µL. The reaction mixture consisted of 1 × KAPA Sybr Fast (Sigma-Aldrich), 500 nM unmodified primer and 1 µL of recovered ssDNA. The thermocycling program consisted of denaturation for 3 min at 95 °C followed by 35 cycles of 15 s at 95 °C, 20 s at 62 °C and 1 s at 72 °C. Fluorescence was acquired at each cycle on the green channel during the elongation step. Determination of the DNA concentration was based on serial dilutions of the ssDNA library (10^3^ -10^8^ molecules/reaction) performed in 500 µg/L poly (dI-dC) (Merck).

Samples were measured undiluted and diluted 1:10 in 10 mM Tris–HCl, pH 8.0. All samples, standards and non-template controls were measured in technical duplicates.

### Monitoring sequence diversity by melting curves

Sequence diversity and sequence enrichment were monitored during the SELEX process by means of melting curves [17, 32]. To this end, 10^5^ DNA molecules were amplified in a total volume of 25 µL. The reaction mixture consisted of 1 × Q5 buffer (NEB), 0.2 mM dNTPs, 1 µM of each unmodified primer, 1 × EvaGreen Plus dye (Biotium) and 0.5 units of Q5 High-Fidelity DNA polymerase (NEB). Reactions were performed on a Mastercycler ep realplex Real-time PCR system (Eppendorf) with the following conditions: denaturation for 3 min at 95 °C followed by 35 cycles of 15 s at 95 °C, 15 s at 55 °C and 15 s at 72 °C. After amplification, a melting profile was applied which consisted of 3 min at 95 °C, followed by 15 s at 95 °C, 15 s at 70 °C and gradual increase (0.03 °C/second) from 70 °C to 90 °C. The whole-cell SELEX process was stopped when melting curves showed a distinct increase of the homoduplex peak, indicating sequence enrichment.

### Identification of candidate aptamers by next-generation sequencing

DNA recovered after SELEX rounds 2, 6, 7, 8 and 9 of SELEX-1 and SELEX-2 and after all rounds of SELEX-3 was prepared for next-generation sequencing. First, the ssDNA was diluted to 10^6^ molecules/µL and 1 µL used to perform a preparative PCR with the optimal number of cycles. Amplified DNA was purified with the Monarch PCR & DNA Cleanup Kit (NEB) and preliminary quality control was performed with a fragment analyzer (Advanced Analytical). DNA concentration was determined using Qubit (Thermo Fisher Scientific). 22 µL (containing at least 20 ng of DNA) of the purified PCR products were sent for sequencing to the Next Generation Sequencing Facility of the Vienna Biocenter Core Facilities. DNA libraries were prepared by ligation and the DNA was sequenced on the Illumina MiSeq using the PE150 Micro kit (300 cycles) in paired-end mode.

Sequencing data analysis was performed using the previously developed Aptaflow script [17]. Additionally, sequences were clustered according to the Levenshtein distance (k-mer = 3) using FASTAptamer 2.0 (https://fastaptamer2.missouri.edu). To speed up the analysis only the first 10 clusters were generated.

Candidate aptamers belonging to different clusters were selected based on their prevalence in the selected round, their appearance in earlier rounds, their absence in the negative selection (R9-T) and their minimum free energy. Minimum free energy was predicted using RNAFold (http://rna.tbi.univie.ac.at//cgi-bin/RNAWebSuite/RNAfold.cgi) selecting DNA parameters (Matthews model, 2004) and 21 °C in the energy parameters and incorporating G–Quadruplex formation into the structure prediction algorithm. In total, 18 candidate aptamers were tested in binding assays.

### Binding assays and determination of aptamer specificity

To test the binding capabilities and the specificity of the selected aptamers, binding assays were performed and the amount of bound ssDNA determined by two independent methods: fluorescence measurements and quantification by qPCR. Conidia of either *A. niger*, *A. tubingensis* or *A. nidulans* and aptamers used for binding assays were prepared as described above. 10^7^ total conidia were centrifuged at 5,000 g for 5 min and resuspended in 100 nM of each aptamer in a total of 100 µL. Competitors were added to each reaction. The same protocol followed during whole-cell SELEX was applied, with the difference that three washes were performed after the incubation. Before elution, recovered conidia-bound ssDNA was resuspended in 100 µL of ultrapure nuclease-free water for qPCR samples or 10 mM Tris–EDTA (pH 8.00) for fluorescent measurements. Blank reactions without conidia and contamination control reactions without aptamers were performed in parallel. For fluorescence measurements, 50 µL of eluted sample were transferred to a 96-well plate and fluorescence was measured at a Tecan Spark reader. Fluorescence was measured after shaking (5 s) with an excitation wavelength of 485 nm, emission wavelength of 520 nm and a gain of 128. Quantification by qPCR was performed as previously described on the ethanol precipitated and recovered ssDNA.

### Aptamer binding affinity determination

Binding affinity curves were obtained by performing the binding assays with 10^7^ total *A. niger* conidia and different starting aptamer concentrations (ranging from 0 to 600 nM) in a reaction volume of 100 µL. Determination of the bound ssDNA was performed by fluorescent measurements at a Tecan Spark reader on 50 µL of the eluted samples. Two replicates were performed for each tested concentration. Values obtained from samples containing only conidia were used as blanks and subtracted from the values obtained in the other samples. Curve fitting to the experimental binding data of the aptamers was done in R (version 4.3.2). Specifically, the nls () function was employed to fit a hyperbolic model ([A]_bound_ ~ [F]) [F] ~ (n * [A])/([A] + K_D_) to the experimental data, enabling the determination of dissociation constant (K_D_) values. 95% confidence intervals for K_D_ were computed using the confint() function in R.

## Results and discussion

### Implementation and design of a whole-cell SELEX process for the development of aptamers specific to *A. niger* conidia

**Fig. 1:**
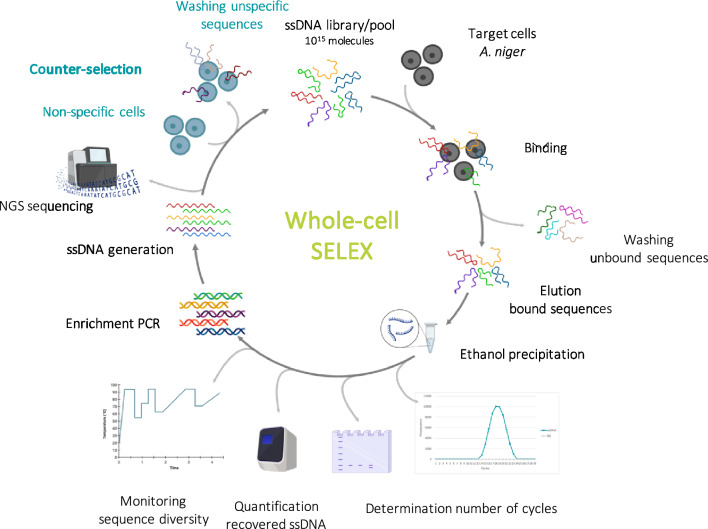
Schematic illustration of the whole-cell SELEX process applied to select aptamers specific to *A. niger* conidia. Nine consecutive rounds and three SELEX experiments were performed. Counter-selection with *A. tubingensis* was introduced after round 4. This figure was partly created with https://www.biorender.com/

A whole-cell SELEX approach was applied to conidia of *A. niger* as illustrated in Fig. 1. Three main changes were introduced to a previously published protocol used to select aptamers against bacterial cells [17]: the overnight precipitation in ethanol to recover the bound-ssDNA after each SELEX round, an additional test-PCR (dnc2) for the determination of the number of cycles and the negative selection performed with *A. tubingensis* before sequencing. Compared to a previous study where aptamers against three different *Aspergillus* species were generated with a toggle approach [26], here we aimed at the *in-vitro* selection of species-specific aptamers. Advanced tools (qPCR and melting curves) were applied to quantify and monitor the diversity of the recovered ssDNA, while next-generation sequencing was used to identify potential aptamer candidates binding to fungal conidia.

In the first round, conidia of the two *A. niger* strains ATCC 1015 and CBS 554.65 were incubated with a total of 10^15^ molecules of the FAM-labelled ssDNA library. Upon incubation, the unbound sequences were removed by centrifugation and washing. The bound sequences were recovered by elution and subsequent precipitation in ethanol to remove the conidia from the recovered ssDNA before PCR amplification. This step was introduced due to the large amounts of PCR inhibitors present in *A. niger* conidia, among which melanin, that would otherwise strongly interfere with the amplification reaction [7, 10, 36]. After recovery, the precipitated ssDNA was subjected to two independent test-PCRs (dnc1 and dnc2) for the determination of the optimal number of cycles for enrichment PCR. This is crucial for the subsequent efficient ssDNA generation via lambda exonuclease. The optimal number of cycles is defined as the number of cycles at which the highest PCR product yield can be obtained without generating by-products [34]. Recovered ssDNA was subsequently amplified by enrichment PCR with the selected number of cycles and single-stranded DNA generated from it with a lambda exonuclease enzyme, before being subjected to another round of *in-vitro* selection. In total, 3 independent whole-cell SELEX experiments were performed, each consisting of a total of 9 rounds. The selection conditions applied in each round are reported in Tables 1, 2 and 3. In general, the conditions were rendered more stringent over the rounds by decreasing the amount of input DNA, increasing the number of washes or adding competitors to the reaction. To increase the species-specificity of the enriched sequences, counter-selection was performed starting from round 4 until round 8. In this case, the recovered ssDNA was incubated first with *A. tubingensis* and the unbound sequences were then recovered by centrifugation and incubated with *A. niger*. This allowed to preferentially enrich sequences that only bind to *A. niger* and do not recognize conidia of its close relative *A. tubingensis*. In all three SELEX experiments, round 9 was performed in parallel against both target (*A. niger*; R9-N) and non-target species (*A. tubingensis*; R9-T) using aliquots of the ssDNA pool recovered after round 8. This allowed to assess unspecific binding to *A. tubingensis* and potential PCR bias introduced during the *in-vitro* selection. Additionally, a positive selection against *A. niger* with counter-selection against *A. tubingensis* was performed in SELEX-3 (R9 in Table 3).

**Fig. 2:**
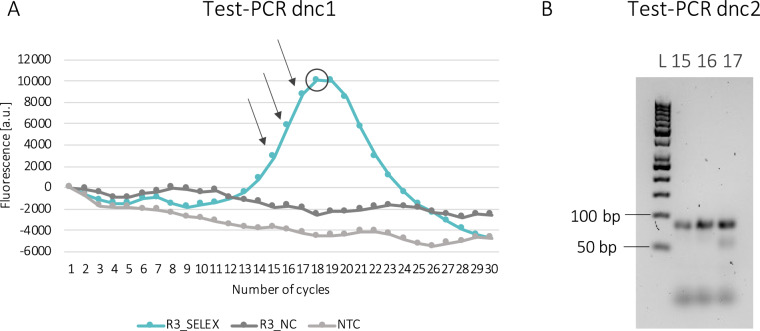
Exemplary results from test-PCRs dnc1 (**A**) and dnc2 (**B**) performed on recovered ssDNA to determine the optimal number of cycles for subsequent amplification. In A, amplification of ssDNA recovered in round 3 of SELEX-3 (R3_SELEX) is shown alongside with the negative control (R3_NC) and the non-template control (NTC). The circle highlights the number of cycles (18) at which the peak of the fluorescence signal is reached while the three arrows point at the number of cycles (15, 16 and 17) before the peak which were chosen for testing in dnc2. In dnc1 the fluorescence signal was normalized to 0. In Figure B, L: ladder, 15, 16 and 17: PCR product after 15, 16 and 17 cycles

When using lambda exonuclease for the generation of ssDNA, it is important that only full-length double-stranded products (homoduplexes) are generated during enrichment PCR, as heteroduplexes cannot be efficiently digested by the enzyme. Due to the high sequence heterogeneity characterizing the utilized random ssDNA library, there is a risk of forming by-products (heteroduplexes) and introducing biases during the PCR [16, 29]. A commonly used method to avoid formation of heteroduplexes is the determination of the optimal number of cycle prior enrichment PCR [25]. To this end, an aliquot of the recovered ssDNA was subjected to two test-PCRs: dnc1 (Fig. 2A) and dnc2 (Fig. 2B). The amplification profile of the ssDNA recovered after round 3 of SELEX-3 is illustrated in Fig. 2A as an example. The fluorescence signal increases over the amplification cycles, before reaching a maximum at cycle 18. The subsequent decrease in fluorescence, previously described as “hook effect”, corresponds to the formation of heteroduplexes [35]. Heteroduplexes, which start to form after depletion of the primers, generally have a lower melting temperature than the full-length PCR products (homoduplexes), as they are composed of only partially complementary sequences. If their melting temperature is lower than the temperature at which the fluorescence signal is measured, they will be dissociated during the measurement. This is reflected in a lower fluorescent signal which causes the hook effect [35]. In a previous study, this test-PCR only was sufficient to determine the optimal number of amplification cycles, corresponding to the number of cycles before the peak [17]. In this study, three reactions were performed in an additional test-PCR (Fig. 2B) with the three subsequent number of cycles before the peak (15, 16 and 17 in the example of Fig. 2A). This allowed to obtain an independent confirmation of the absence of heteroduplexes after the enrichment PCR. In the case reported in Fig. 2B, the cycle right before the peak (17) corresponded to the start of heteroduplex formation, visible as a shorter product on the gel, while after 16 cycles only the specific product (86 bp, homoduplexes) was visible. This led to the decision of using 16 cycles to perform amplification of the recovered ssDNA.

### Monitoring the amount and the diversity of the recovered ssDNA during the SELEX process

**Fig. 3:**
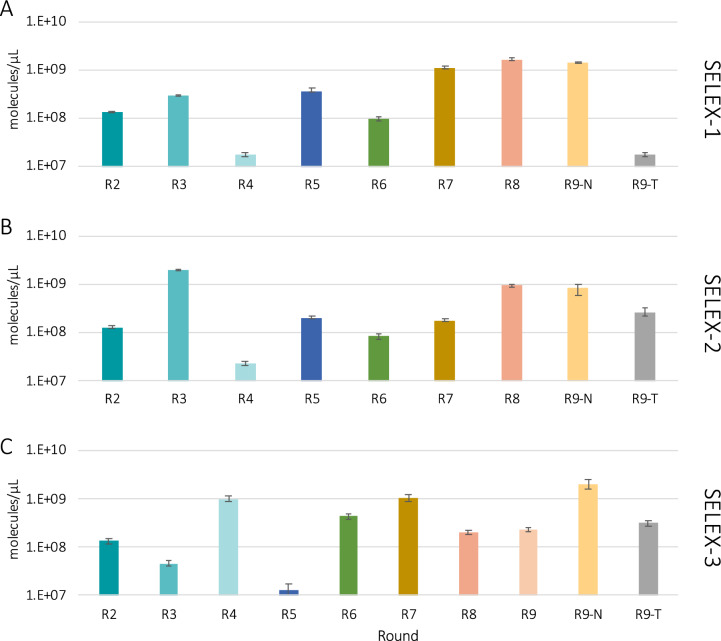
Quantification by qPCR of the ssDNA recovered after each round in the three SELEX experiments (**A**: SELEX-1, **B**: SELEX-2 and **C**: SELEX-3). R2 to R9-T refer to the specific rounds of SELEX-1, SELEX-2 and SELEX-3 described in Tables 1, 2 and 3, respectively. Error bars indicate min. and max. values

**Fig. 4:**
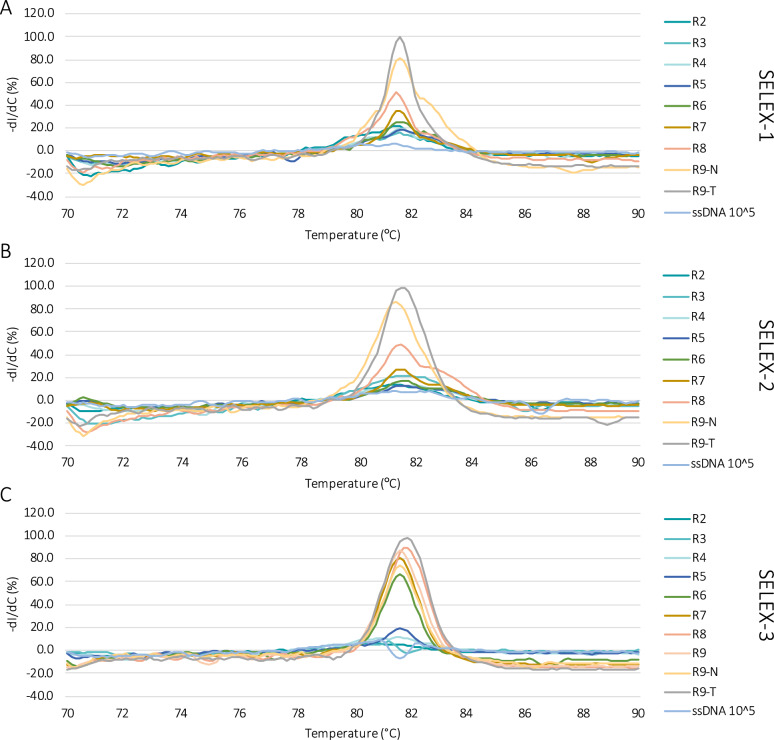
Melting curves of the ssDNA recovered after each round in the three SELEX experiments (**A** : SELEX-1,** B** : SELEX-2 and** C**: SELEX-3). R2 to R9-T refer to the specific rounds of SELEX-1, SELEX-2 and SELEX-3 described in Tables 1, 2 and 3, respectively. Melting curve of 10^5^ molecules of the original ssDNA library (ssDNA 10^5) was performed as comparison

Advanced tools were applied to monitor the *in-vitro* selection process allowing to precisely quantify the amount by qPCR (Fig. 3) and measure changes in the diversity by melting curves of the recovered ssDNA after each round (Fig. 4). Round 1 was excluded from these analyses as all of the recovered ssDNA was amplified for further processing.

Quantification of the recovered ssDNA was performed by qPCR. With this method, absolute recovered DNA quantities can be determined with high sensitivity [1]. Differences in the amount of recovered ssDNA could be observed between different rounds, with concentrations ranging from 10^7^ to 10^9^ molecules/µL.

The increase in the amount of bound DNA was reported in literature as an indicator of successful sequence enrichment [16]. However, the amount of recovered DNA does not only depend on the enrichment of certain sequences but also on the selection conditions applied at each round and on the specificity and accessibility of conidia surface targets to the binder sequences present in the ssDNA pool. The decrease of recovered DNA measured at round 4 of SELEX-1 and SELEX-2 might be due to the counter-selection, which was applied starting from this round. However, a similar effect is not visible in SELEX-3, indicating that it is likely a combination of factors, rather than one factor only, to contribute to the number of bound sequences. Moreover, although all the three SELEX experiments were initiated with the same ssDNA library, the sequences randomly present in each aliquot were not the same and most likely led to different enrichment patterns. Round 5 of SELEX-3 yielded a very low amount of DNA (Fig. 3A) and in order to obtain enough DNA to continue with round 6 of the SELEX process, additional DNA was obtained by the dilution and further amplification of the already amplified PCR product, introducing a bias in the selection.

When the same ssDNA pool was applied to *A. niger* (R9-N) or to *A. tubingensis* (R9-T), higher concentrations of recovered ssDNA were measured for R9-N in all SELEX experiments. This suggests successful enrichment of *A. niger*-specific sequences.

A more effective method to monitor sequence enrichment is based on the analysis of the melting curves, performed on the recovered DNA after PCR amplification [17, 32]. Melting curves of the three SELEX experiments are reported in Fig. 4. Melting curves allow to monitor the formation of homoduplexes, derived from the annealing of two complementary strands of a PCR products. At the beginning of selection, homoduplexes are rare, as most of the sequences are unique. However, if sequence enrichment is successful, homoduplex formation can be observed as a distinct melting peak at around 82 °C in the melting profile. Distinct melting peaks started to appear in rounds 7 of SELEX-1 and SELEX-2 and in round 5 of SELEX-3, indicating a decrease in sequence diversity and the appearance of enriched sequences. Melting peaks increased further in subsequent rounds, suggesting further enrichment. However, while this increase appeared gradual in SELEX-1 and SELEX-2, it was abrupt between rounds 5 and 6 of SELEX-3. This is most likely due to the PCR bias introduced in this experiment which led to the loss of sequences present in low abundance while those present in higher copies had a higher chance to be amplified and carried over to the next round. Different peak shapes correspond to changes in nucleotide composition of the analyzed pool [17]. Based on the evolution of the melting peaks, the selection was stopped after nine rounds. In SELEX-3, the highest melting peak was reached at round 8, suggesting a loss of potential binders at round 9. Interestingly, melting peaks of round 9-T (selection against *A. tubingensis* in round 9) were higher than those of round 9-N.

### NGS data analysis and selection of aptamer candidates

**Fig. 5:**
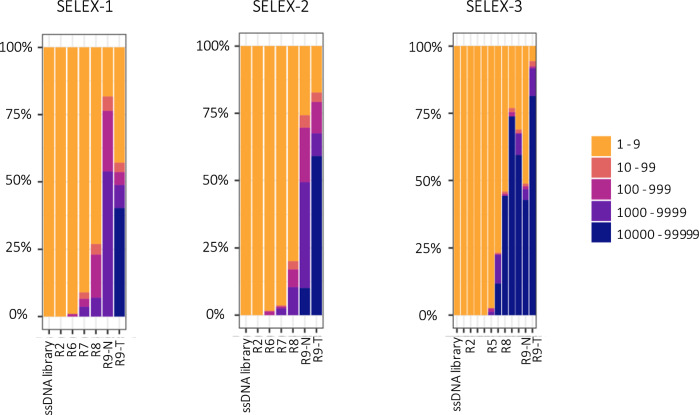
Total count of individual sequences over the rounds shown in percentage. Sequencing was performed on ssDNA pools recovered after rounds 2, 6, 7, 8, 9-N and 9-T of SELEX-1 and SELEX-2 and after all rounds of SELEX-3. Additionally, the initial ssDNA library was sequenced to check for biases. R2 to R9-T refer to the specific rounds of SELEX-1, SELEX-2 and SELEX-3 described in Tables 1, 2 and 3, respectively

Sequencing data were processed with the previously developed Aptaflow script [17]. Graphs showing the sequence enrichment in recovered ssDNA pools over the subsequent SELEX rounds (Fig. 5) and a list of the 1000 most enriched sequences for each round were generated. The total count of individual sequences, representing sequence enrichment, increased during the subsequent rounds in all three performed SELEX experiments, reaching a peak at round 9 in SELEX-1 and SELEX-2 and at round 8 in SELEX-3. The sequencing data confirmed the changes in diversity observed in the melting curves, highlighting the power of combining these two techniques to determine how many SELEX rounds to perform and which rounds to sequence. As already observed in the melting curves, the increased count of enriched sequences in round 9-T (negative selection, performed with *A. tubingensis* instead of *A. niger*) might indicate that the incubation of the aptamers with *A. tubingensis* after selection with *A. niger* led to a loss of diversity of specific enriched sequences. Only a few sequences were retained by *A. tubingensis* conidia and these had a higher chance to be amplified at higher rates. This phenomenon is reflected as an apparent increase in sequence enrichment, similar to what observed in round 6 of SELEX-3, which, however, does not correspond to an increase of binder molecules. Therefore, performing negative selection and subsequent analysis of the sequences enriched in such an unspecific round can be a valuable strategy to more easily identify potential binders enriched in the target rounds as well as to remove unspecific sequences from the potential binding candidates.

The first ten selected aptamer candidates were identified from round 8 of SELEX-3, as melting curve analyses, as well as the sequencing data, showed the highest enrichment during this round. Additional 8 potential aptamer candidates were identified from rounds 9 of SELEX-1 and SELEX-2. Selection was performed on sequences belonging to different clusters, by ranking them based on their prevalence at the selected round and their appearance in earlier rounds. Sequences AN03-R8-AN435, AN03-R9-N-AN070, AN01-R9-095, AN01-R9-105, AN01-R9-115, AN02-R9-099 and AN02-R9-185 were selected because present with higher reads in the positive selection round (R9-N) than in the negative selection round (R9-T). AN03-R8-AN156 was selected as negative control as it showed higher read counts in round 9-T than in any of the rounds performed with *A. niger* conidia as targets. The minimum free energy and the secondary structure of the selected sequences were predicted using the online tool RNAFold 2.5.1. All 18 selected aptamer candidates with their characteristics and reason for selection are listed in Supplementary Table 1.

### Aptamer identification and impact of the FAM label on the aptamer binding

The 18 selected aptamer candidates were screened for their capability to bind to *A. niger* conidia by performing binding assays and subsequent quantification of the recovered ssDNA by qPCR.

**Fig. 6:**
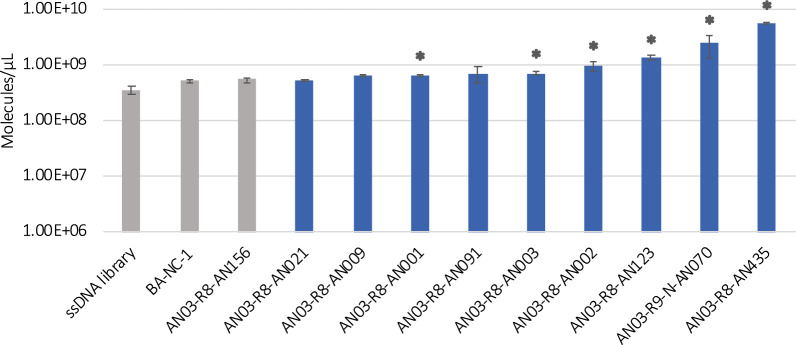
First screening of FAM-labelled candidate aptamers. Recovered ssDNA after binding assays with *A. niger* conidia was quantified by qPCR and is shown in molecules/µL. Samples were measured in duplicates. The mean value between biological and technical replicates is displayed after subtracting the mean value of the blank samples (only aptamers, no conidia). Error bars represent max. and min. values. Asterisks indicate aptamers showing significantly different binding (p-value < 0.05) compared to the negative controls (ssDNA library and BA-NC-1)

In a first screening experiment, ten of the selected candidates were ordered with a FAM-label at the 5´end (Fig. 6). The quantified DNA was compared to three negative controls: the starting ssDNA library, a labelled random negative control (BA-NC-1) and the AN03-R8-AN156 sequence.

**Fig. 7:**
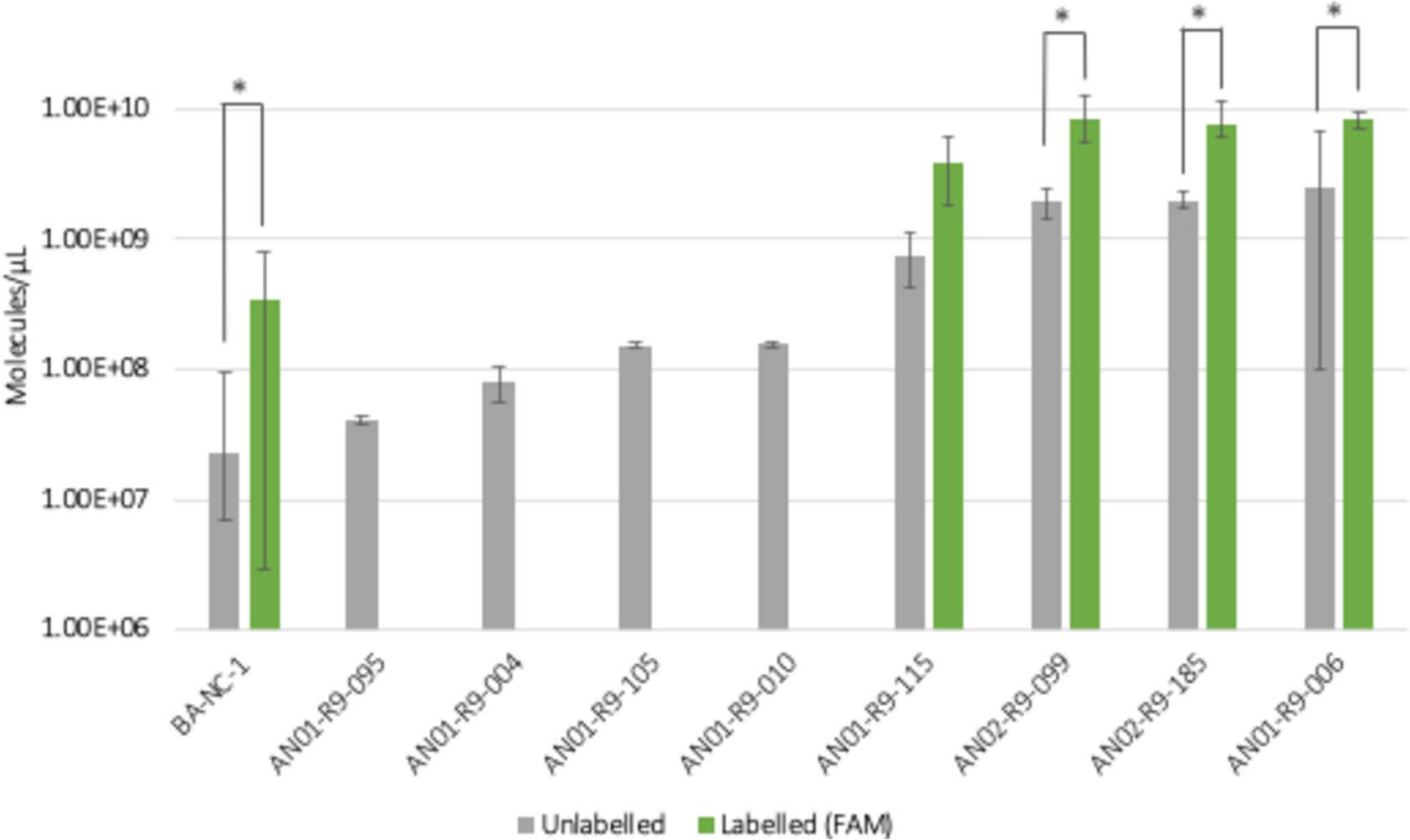
Second screening of FAM-labelled and unlabeled candidate aptamers. Recovered ssDNA after binding assays with *A. niger* conidia was quantified by qPCR and is shown in molecules/µL. Samples were measured in technical duplicates. The mean value between biological and technical replicates is displayed after subtracting the mean value of the blank samples (only aptamers, no conidia). Error bars represent max. and min. values. Asterisks indicate aptamers showing significantly different binding (p-value < 0.05) between unlabelled and labelled version

The ssDNA recovered after most of the binding assays was similar to the amount of ssDNA recovered after incubation of *A. niger* conidia with either the ssDNA library, the negative control BA-NC-1 or the negative control AN03-R8-AN156. This might be due to the PCR bias introduced in round 6 of SELEX-3, which most likely led to the enrichment of sequences which are more easily amplified but might not bind to the conidia and the concomitant loss of potential binders. However, two candidates, AN03-R8-AN435 and AN03-R9-N-AN070, showed significantly higher recovery rates compared to the negative controls, indicating that they can bind to the target conidia. As a high background could be measured for the negative controls (ssDNA library, BA-NC-1 and AN03-R8-AN156), a second screening was performed with unlabeled aptamer candidates to determine if the FAM-label had an effect on the binding process. To this end, eight aptamer candidates identified in SELEX-1 and SELEX-2 were tested in their unlabeled version and compared to the unlabeled BA-NC-1 (Fig. 7). Additionally, labelled and unlabeled versions of aptamer candidates AN01-R9-006, AN01-R9-115, AN02-R9-099 and AN02-R9-185 and of the negative control BA-NC-1 were compared (Fig. 7). Candidates AN01-R9-006, AN01-R9-115, AN02-R9-099 and AN02-R9-185 showed higher recovery rates than the negative control in both versions (labelled and unlabeled). Interestingly, labelled aptamers were associated with higher recovery rates than unlabeled ones. We confirmed that this was not an artifact due to the interference of the FAM fluorescence during the qPCR, but it rather derived from higher binding of the FAM-labelled sequences to conidia of *A. niger* than the unlabeled counterparts. This suggests that the FAM fluorophore itself interacts with the target cells to a certain extent. To our knowledge, FAM is not used as a cell or cell wall dye but mostly conjugated to a DNA or an RNA oligo. Based on the measured recovery rates, aptamers AN03-R8-AN435, AN03-R9-N-AN070, AN01-R9-006, AN01-R9-115, AN02-R9-099 and AN02-R9-185 were selected for further characterization.

### Aptamer specificity to other *Aspergillus* species

**Fig. 8:**
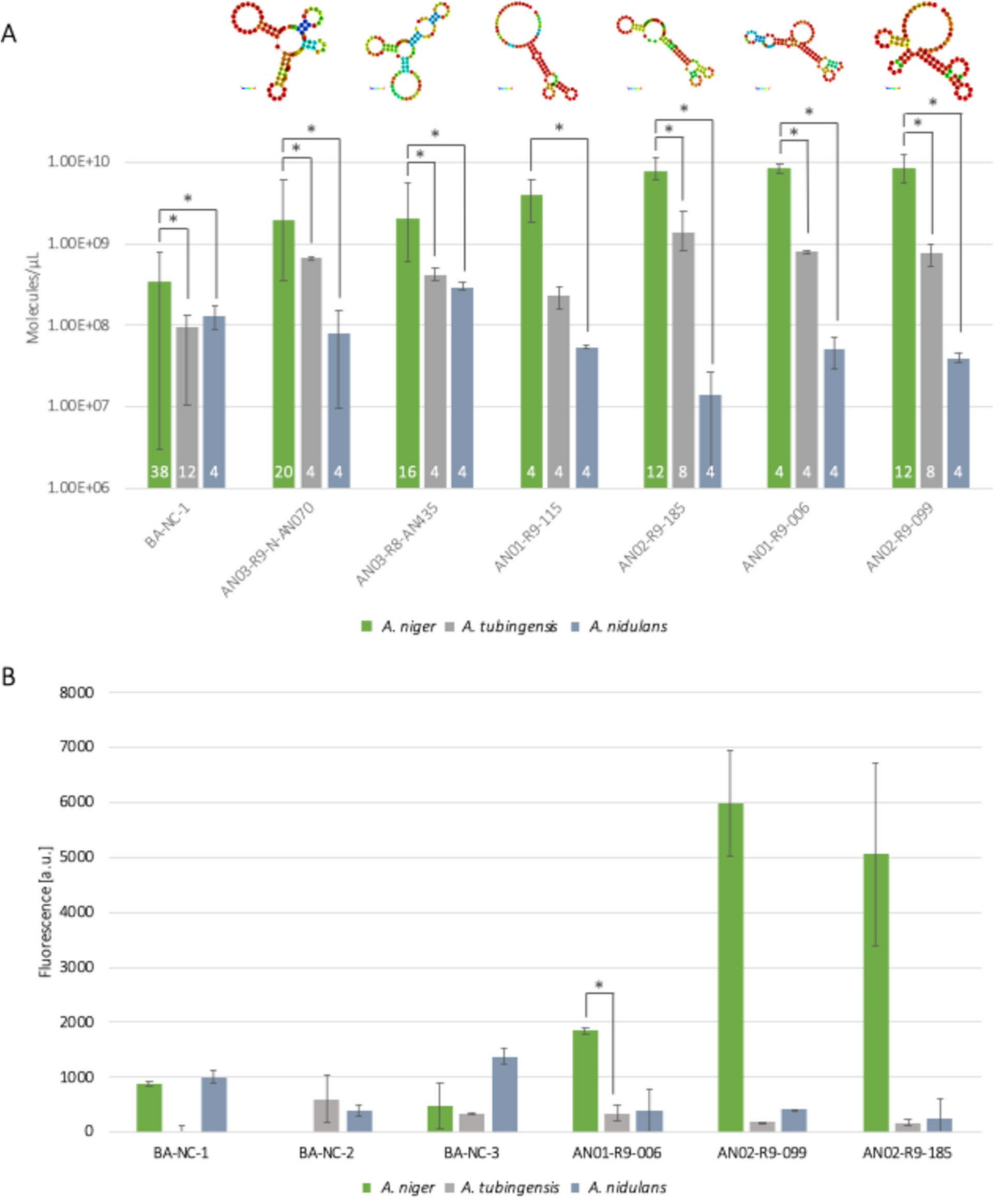
Specificity evaluation of selected aptamers by means of qPCR (**A**) or fluorescence (**B**) measurements. **A**: recovered ssDNA after binding assays with *A. niger*, *A. tubingensis* and *A. nidulans* conidia was quantified by qPCR and is shown in molecules/µL. Samples were measured in technical duplicates. The mean value between biological and technical replicates is displayed after subtracting the mean value of the blank samples (only aptamers, no conidia). The number of biological and technical replicates performed is indicated in white in each bar. Error bars represent max. and min. values. The predicted secondary structure of each aptamer was obtained with RNAFold and is shown on top of the figure. **B**: confirmation of the species-specificity of three selected aptamers by fluorescent measurements upon elution. Fluorescence of the recovered and eluted ssDNA after binding assays with *A. niger*, *A. tubingensis* and *A. nidulans* conidia was measured at a Tecan Spark reader. Samples were measured in duplicates. The mean value between biological and technical replicates is displayed after subtracting the mean value of the blank samples (only conidia, no aptamer). Error bars represent max. and min. values. Asterisks indicate aptamers showing significantly different binding (p-value < 0.05) between different strains

To determine whether the six selected aptamers can bind to *A. niger* in a species-specific manner, binding assays were performed with other two *Aspergillus* species, *A. tubingensis* and *A. nidulans*. Based on the qPCR results, all the selected aptamers showed to be species-specific for *A. niger* (Fig. 8A). DNA recovered after incubation with *A. niger* increased from 2.5 to 17-fold when compared to *A. tubingensis* and from 7 to 500-fold when compared to *A. nidulans*. Interestingly, the negative control (BA-NC-1) seems to bind preferentially to the conidia of *A. niger* than to those of the other two fungal species. This sequence was not present in the sequencing data but it was randomly generated and it is possible that it binds to a certain extent to the conidia of *A. niger.*

To confirm successful and species-specific binding, fluorescent measurements were performed on the eluted samples upon binding with three selected aptamers (AN01-R9-006, AN02-R9-099 and AN02-R9-185) (Fig. 8B). Additionally, to avoid the introduction of a bias due to the selection of the random sequence, other two negative controls differing in the unique internal 40 bp region (BA-NC-2 and BA-NC-3) were measured in parallel (Fig. 8B).

Fluorescent measurements confirmed the species-specific binding of the selected aptamers to *A. niger* conidia. Furthermore, different fluorescent values could be measured when comparing the three different negative controls. The first selected negative control (BA-NC-1) showed the highest binding to *A. niger* conidia. BA-NC-2 did not bind at all to target conidia and BA-NC-3 only slightly. Therefore, randomly selected sequences have the potential to bind to a certain extent to the target cells. These results highlight the importance of choosing a suitable negative control and suggest that using multiple negative controls should be preferred.

*A. niger* and *A. tubingensis* are phylogenetically closely related, belonging both to the section *Nigri* of the genus *Aspergillus* [33]. Due to their highly similar phenotype, they can be hardly distinguished based on classical morphological criteria and the use of molecular analyses is crucial for their differentiation [22, 27]. The capability of the DNA aptamers developed in this study to distinguish between these closely related species is of high relevance and indicates that these fungi might substantially differ in their surface proteome. These results could open the way to new strategies in the identification and characterization of closely related *Aspergillus* species.

### Aptamer binding affinity

**Fig. 9:**
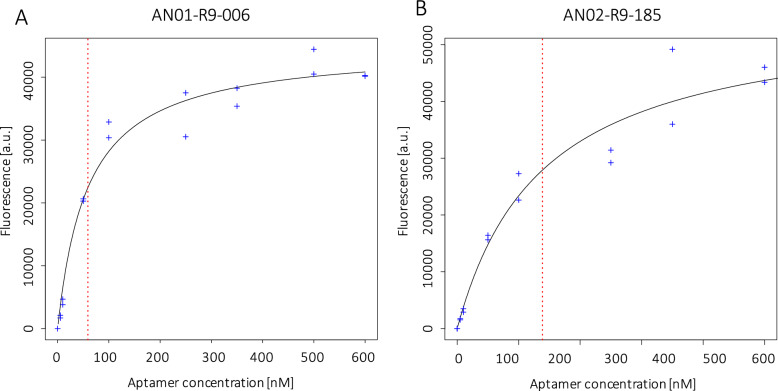
Binding affinity of aptamers AN01-R9-006 (A) and AN02-R9-185 (B) to *A. niger* conidia. Single measurements are indicated by blue crosses and the corresponding fitted curve is shown as a black line. The red dotted line corresponds to the K_D_ value

The binding affinity of the aptamers AN01-R9-006, AN02-R9-099 and AN02-R9-185 was determined by incubating the *A. niger* conidia with different concentrations of the corresponding aptamer. The binding curves were obtained by measuring fluorescence after elution (Fig. 9).

The binding curve of aptamer AN02-R9-099 did not show saturation (data not shown), indicating that this aptamer might bind non-specifically to the conidia of *A. niger* [14]. K_D_ values were calculated for aptamers AN01-R9-006 and AN02-R9-185. AN01-R9-006 showed a K_D_ of 58.97 nM (95% confidence interval 42.89–81.03 nM). AN02-R9-185 showed a K_D_ of 138.71 nM (95% confidence interval 79.65—255.51 nM). The measured equilibrium dissociation constants are in the range of aptamers previously developed against fungal conidia [26] and indicate specific binding on the conidial surface with high affinity. The binding affinity curves indicate that the aptamers interact in a concentration-dependent manner with the *A. niger* conidia.

## Conclusions

In this study, whole-cell SELEX was optimized for conidia of *A. niger*. Three independent and parallel whole-cell SELEX experiments increased the likelihood of selecting and identifying effective binders. Two independent test-PCRs were performed at each round, which allowed to control for potential biases arising from PCR amplification. Quantification and monitoring of the diversity of the recovered ssDNA allowed to obtain insights into the SELEX process before sequencing. Next-generation sequencing was performed on the obtained enriched ssDNA pools, allowing the identification of sequences binding with high affinity to *A. niger* conidia. By introducing counter-selection steps and a negative selection against the closely related *Aspergillus* species *A. tubingensis*, species-specific aptamers could be obtained. The binding affinity to *A. niger* conidia of two of the developed aptamers, AN01-R9-006 and AN02-R9-185, was determined to be 58.97 and 138.71 nM, respectively. The protocol reported here could be easily transferred to conidia of other fungi, allowing the development of aptamers against any *Aspergillus* species.

The availability of DNA molecules able to distinguish closely related fungal species creates new opportunities in fungal research. DNA aptamers could be used to better understand the complex structures constituting the external surface of fungal conidia. Not only the developed aptamers could be implemented as biosensors for quantitative monitoring and detection of fungal conidia, but their species-specificity feature could be exploited for the rapid identification of morphologically identical *Aspergillus* species in various fields, from clinical to taxonomical applications. To this end, future work should focus on the thorough characterization the identified aptamers. Determination of their binding properties, including binding assays with multiple strains and identification of the aptamers´ cellular target(s), is crucial to allow for reproducible results and, in the end, successful application of the identified molecules.

## Supplementary Information

Additional file 1.

## Data Availability

No datasets were generated or analysed during the current study.
