# Supplementary material for: Development of a whole-cell SELEX process to select species-specific aptamers against Aspergillus niger
Source: Fungal Biol Biotechnol. 2024 Nov 5;11:17. doi: 10.1186/s40694-024-00185-2 (PMC11536964; doi:10.1186/s40694-024-00185-2)
Supplement: Supplementary file 1 — Additional file 1. [file 40694_2024_185_MOESM1_ESM.pdf]

Supplementary Table 1: Characteristics of selected aptamers for screening

Name	40 bp unique sequence [5´-> 3´]	SELEX	Rank	First appearance	Cluster	Minimum free energy secondary structure [kcal/mol]	Comment
AN03-R8-AN001	GATGGTGGCGTGCTGCAAGGATTTCCCATTTTTCATTGGC	3	R8-AN03: 1	ssDNA library	R8: 1	-9.98	In top 10 AN03-R8, early appearance
AN03-R8-AN002	CCGGTCATTTGTCTAAGCGGCGTTCCGATAGGGATTTCCT	3	R8-AN03: 2	ssDNA library	R8: 2	-14.10	In top 10 AN03-R8, early appearance
AN03-R8-AN003	GAGTGGCGTCAGTTTCGAACAGAAACGCTGTCCTCAACTT	3	R8-AN03: 3	AN03: R1	R8: 3	-14.92	In top 10 AN03-R8, early appearance
AN03-R8-AN009	GATGGTGGCGTTCTGCAAGGATTTCCCATTTTTCATTGGC	3	R8-AN03: 9	AN03: R5	R8: 4	-10.53	In top 10 AN03-R8, early appearance
AN03-R8-AN021	GGTGGCGTTCTATCCAAGGATTTCCCACATATTACCATTC	3	R8-AN03: 21	AN03: R5	R8: 5	-6.55	In top 10 AN03-R8, early appearance
AN03-R8-AN091	ACTCCCCCCGTGTTACATGGGGCGGCTTCTGCATATCAGT	3	R8-AN03: 91	AN03: R5	R8: 6	-11.32	High rank and different cluster
AN03-R8-AN123	ACGCTGTCCAGGGTGGCGCTCAGAATCACCTTCTCACTTA	3	R8-AN03: 123	AN03: R5	R8: 7	-13.20	High rank and different cluster
AN03-R8-AN435	CACCACCACGACACACAACCTTCCCGTGCGGACCCAGCGA	3	R8-AN03: 435	AN03: R8	R8: >10	-7.69	R9-N > R9-T
AN03-R9-N-AN070	TCCCAGCGCCCGGAGAACACGAGGAACGCACCTATCACAC	3	R9-N-AN03: 70	AN03: R9-N	R9: >10	-11.63	R9-N > R9-T
AN03-R8-AN156	CCGGTCATTTGTCTAAGCGGCTTTCCGATAGGGATTTCCT	3	R8-AN03: 156	AN03: R7	R8: 2	-12.09	R9-N > R9-T, negative control
AN01-R9-004	CCAGACTAGTTCGCCTCCACGAAGGGATTAAATCCAATCC	1	R9-AN01: 4	AN01: R7	R9-AN01: 4	-12.30	In top 10 AN01-R9
AN01-R9-006	CCGGATGCTCTACCGTACTAGTACGACTCCACGAAATTAT	1	R9-AN01: 6	AN01: R6	R9-AN01: 6	-13.73	In top 10 AN01-R9
AN01-R9-010	CCATATCGCTGACCTACTAGTACGCCTCCACGAAGTCCCG	1	R9-AN01: 10	AN01: R7	R9-AN01: 10	-13.41	In top 10 AN01-R9
AN01-R9-095	TCGTAACGTCTCCACGAACTAGCTTGGACGATATCAGCCT	1	R9-AN01: 95	AN01: R7	R9-AN01: >10	-10.3	Absent or low in AN01-R9-T
AN01-R9-105	ACATGGACGAGTACGCCTCCACGAAGCGGTCCCCTAACCG	1	R9-AN01: 105	AN01: R7	R9-AN01: >10	-13.8	Absent or low in AN01-R9-T
AN01-R9-115	CCGACATCTTTGTACTAGTACGCCTCCACGAAAACACACT	1	R9-AN01: 115	AN01: R7	R9-AN01: 9	-15.45	Absent or low in AN01-R9-T
AN02-R9-099	CCTGAGTAACTGCTCGTACTAGTTCGCCTCCTCGAATTAC	2	R9-AN02: 99	AN03: R9	R9.T-AN02: > 10	-15.56	Absent or low in AN02-R9-T
AN02-R9-185	ACTTCGCAGTCTGACTAGTACGCCTCCACGAAGGGTTTCT	2	R9-AN02: 185	AN03: R9	R9.T-AN02: > 10	-12.12	Absent or low in AN02-R9-T
